# 2401: Professional Mentoring Skills to Enhance Diversity (PROMISED): Diversifying the workforce

**DOI:** 10.1017/cts.2017.178

**Published:** 2018-05-10

**Authors:** Doris Rubio, Marie Norman, Seyed Mehdi Nouraie, Shanta Zimmer, Brian Primack, Esa Davis, Jeanette South-Paul

**Affiliations:** University of Pittsburgh, Pittsburgh, PA, USA

## Abstract

OBJECTIVES/SPECIFIC AIMS: To diversify the workforce by providing leadership and career coaching training to mentors so that they can be better leaders with their trainees and incorporate career coaching skills into their mentoring style. METHODS/STUDY POPULATION: PROMISED Program helps current and future members of the National Research Mentoring Network (NRMN) develop management, leadership, and career coaching skills so that they may be more effective in guiding their mentees. Studies show that mentees remain engaged in research when they drive their own careers, but mentors rarely help them recognize ways to do this. PROMISED aims to address by providing online leadership training and career coaching training. We developed innovative online leadership training for mentors committed to mentoring people from diverse backgrounds that are focused on management and leadership skills. These modules contain exercises, self-assessments, and discussion boards. We also have reading materials and other supplemental work such as videos to augment the modules. We also created 2-day training on career coaching skills for mentors. Certified career coaches trained participants in career coaching tools so that they could incorporate these skills into their mentoring style. Mentors tend to view themselves as content advisors, and they focus on the next step in the research project rather than the research career. We trained mentors to provide career coaching to their mentees, which will help the mentee establish a successful biomedical research career trajectory. RESULTS/ANTICIPATED RESULTS: In total, 45 mentors attended the Career Coaching Workshop. We assessed 26 mentoring/career coaching traits. Every trait improved on post survey (Likert scale 1–7), for example, “Taking into account the biases and prejudices you bring to the mentor/mentee relationship” (Pre: 4.16, Post: 5.38) and Working with mentees to set clear expectations of the mentoring relationship (Pre: 4.27, Post: 5.32). Some comments from attendees included: “amazing,” “powerful,” “excellent program,” “learned so much.” For the online module, we have a maximum of 20 fellows enrolled in each module. Results show that the fellows rate the module extremely useful. A comment from 1 fellow confirms this: “This session has changed my life and I know that the PROMISED program will transform my abilities as a mentor and as a person.” DISCUSSION/SIGNIFICANCE OF IMPACT: Providing Career Coaching Training and Online leadership skills can significantly improve mentors ability to mentor people, particularly those from diverse backgrounds. In addition, this training can help mentors who are committed to mentoring people from diverse backgrounds promote their own careers as well as their mentees.

